# Knowledge and Awareness of Osteoporosis and Its Risk Factors Among the Adult Population in Jeddah, Saudi Arabia: An Analytical Cross-Sectional Study

**DOI:** 10.7759/cureus.65995

**Published:** 2024-08-02

**Authors:** Lutf A Abumunaser, Ibrahim L Abumunaser, Raed M Sharaf, Qusai Kabouha

**Affiliations:** 1 Department of Orthopedic Surgery, Faculty of Medicine, King Abdulaziz University Hospital, Jeddah, SAU; 2 College of Medicine, Faculty of Medicine, King Abdulaziz University, Jeddah, SAU

**Keywords:** middle east, prevention, healthy lifestyle, underdiagnosis, skeletal disease

## Abstract

Background: Osteoporosis is a medical condition that affects the bones, causing them to become weak and fragile. People with osteoporosis are at a higher risk for fractures, resulting in an increase in morbidity and mortality rates. Despite its high prevalence worldwide, osteoporosis remains underdiagnosed and undertreated, leading to significant public health concerns, especially in Saudi Arabia.

Material and methods: An analytical cross-sectional study was conducted using an online questionnaire to assess the level of awareness of osteoporosis and its risk factors among the adult population in Jeddah, Saudi Arabia. The questionnaire was distributed to a diverse, representative sample of 232 adults in Jeddah, covering demographic data, knowledge and awareness of osteoporosis and its risk factors, and lifestyle practices related to prevention and management. Data were analyzed using IBM Corp. Released 2013. IBM SPSS Statistics for Windows, Version 22.0. Armonk, NY: IBM Corp., employing descriptive analysis, cross-tabulation, and chi-square tests to evaluate knowledge levels and associated factors.

Results: A total of 232 eligible adults participated, with a mean age of 35.6 ± 8.7 years. Most participants, 228 (98.3%), had heard of osteoporosis, and 137 (59.1%) had good overall knowledge of the disease. Key risk factors identified included calcium deficiency (210, 90.5%), increasing age (171, 73.7%), and family history (136, 58.6%). Although most participants accurately identified common risk factors, only 10.8% of participants consistently practiced a healthy and active lifestyle, highlighting the gap between awareness and preventive behaviors. Our study showed that gender (p-value = 0.049), educational level (p-value = 0.044), and whether the participant was diagnosed with or knew someone diagnosed with osteoporosis (p-value = 0.045) were statistically significant factors associated with the overall level of knowledge of osteoporosis in the participants.

Conclusions: This study emphasized the need for targeted interventions to improve knowledge and promote healthier lifestyle habits among adults in Jeddah to reduce the burden of osteoporosis. Future efforts should focus on bridging the gap between awareness and preventive actions, emphasizing the importance of healthy lifestyle habits and knowledge of osteoporosis risk factors.

## Introduction

Osteoporosis is a systemic metabolic skeletal disease characterized by a progressive reduction in bone mass and microarchitectural deterioration of bone tissue, leading to an increase in bone fragility and susceptibility to fracture [1]. The hip, spine, and wrist were the most common sites for fragility fractures attributed to osteoporosis [2]. The one-year mortality rate associated with hip fractures in Saudi Arabia was 27%, which was significantly high when compared to the regional average (17.9%) [3]. Osteoporosis is generally more common among women due to hormonal changes with increasing age; however, its incidence among men is increasing, particularly among older men [1]. According to epidemiological data, the highest prevalence was recorded in Asia (24.3%), followed by Europe and America (16.7% and 11.5%, respectively) [4]. According to a systematic review and meta-analysis, the worldwide prevalence of osteoporosis among older women is 35.3%, which is much higher than that among older men (12.5%) [4]. Furthermore, the prevalence of this disease in the Middle East is 24.4%, with Saudi Arabia having the highest prevalence and Kuwait having the lowest [4]. Additionally, as concluded by Mohammed Bakir et al. in their study on 951 Syrian women, 23.68% were diagnosed with osteoporosis in the lumbar spine, while 13.1% had evidence of osteoporosis in the neck of the femur [5].

Nonmodifiable risk factors include sex, age, race, premature menopause, history of previous fractures, and lactose intolerance [6]. While the aforementioned risk factors are unmodifiable, we can optimize others, such as smoking, alcohol consumption, inadequate calcium and vitamin D consumption, and long-term use of corticosteroids, to reduce the risk of osteoporosis [6,7].

Although treatment options are available and widely approved, prevention remains the most appropriate method for managing this disease and its deleterious complications, the most important of which are fractures [8]. Preventive strategies are mainly based on controlling and managing these modifiable risk factors [8]. A healthy diet that contains fruits, vegetables, animal proteins, and dairy products rich in calcium and vitamin D will enrich the bones with the nutrients needed to protect them and keep them healthy [8]. Furthermore, routine physical activity, including weight-bearing exercises rather than a sedentary lifestyle, will strengthen the muscles, provide more support to the bones and joints, and help decrease the risk of falls [8].

Despite its high prevalence, osteoporosis remains underdiagnosed and undertreated, leading to a higher incidence of fractures, especially among women. Approximately 70% of women aged >65 years diagnosed with osteoporosis in the United States of America (USA) did not receive treatment [9,10]. Furthermore, estimates suggest that 70% of men with osteoporosis do not receive treatment [11]. Interestingly, a cross-sectional study that included 388 female hospital employees and assessed their knowledge about smoking-related illnesses concluded that a high percentage of women were unaware that smoking is a risk factor for developing osteoporosis [12].

Various studies have concluded that there is a significant lack of awareness among the general population and healthcare workers regarding osteoporosis and its risk factors, given its importance and impact on patient's lives and the healthcare system due to the burden of the disease and its complications, especially fractures [12,13]. Therefore, this study aimed to assess the level of awareness of osteoporosis among the adult population of Jeddah, Saudi Arabia.

## Materials and methods

Study design

An analytical cross-sectional study was conducted using an online questionnaire for data collection. The questionnaires were distributed to diverse groups of participants to ensure a representative sample size. The data were analyzed using various statistical methods to identify patterns or trends.

Study area

This study was conducted in the Western Region of the Kingdom of Saudi Arabia, specifically in Jeddah, between November 2023 and April 2024.

Study population

The target population for our study was adults from the general population of Jeddah. To determine our sample size, "Raosoft" was employed. A minimum sample size of 385 people was taken from Jeddah's entire population, including ordinary residents who did not match the requirements for this study, considering a 5% margin of error, a 95% confidence level, and a 50% response distribution. The sample size was calculated from Jeddah's entire population, including residents who didn't fulfill the inclusion criteria of this study.

Inclusion and exclusion criteria

The inclusion criteria for participation in the study were all adults aged 18 years or older and currently residing in Jeddah, Saudi Arabia. The exclusion criteria included children under the age of 18, residents who weren't able to access the online questionnaire, and anyone not residing in Jeddah.

Sampling technique

This study aimed to include individuals from a target population to obtain volunteer responses. To achieve this, a convenience sampling technique was employed, in which participants were selected based on their availability and willingness to participate. Participants were recruited through various channels, such as social media, email invitations, and word-of-mouth referrals. We made efforts to ensure the sample was diverse and representative of the target population in terms of age, sex, and socioeconomic status.

Data collection

Participants were recruited through various channels, such as social media platforms such as Twitter, Facebook, and WhatsApp, email invitations, and word-of-mouth referrals using an online questionnaire. We made efforts to ensure the sample was diverse and representative of the target population in terms of age, sex, and socioeconomic status. The questionnaire used was modified based on a study done among Saudi adults in Riyadh in 2016 [14]. It was available in Arabic and English and comprised three sections. The first section focused on demographic data; the second assessed knowledge and awareness of osteoporosis and its modifiable and nonmodifiable risk factors; and the third assessed osteoporosis prevention and management practices among Jeddah's adult population. The duration of data collection was six months. 

Statistical analysis

After the data were extracted, they were revised, coded, and fed to IBM Corp. Released 2013. IBM SPSS Statistics for Windows, Version 22.0. Armonk, NY: IBM Corp. All statistical analysis was done using two-tailed tests. A p-value less than 0.05 was statistically significant. With regard to knowledge and awareness, each correct answer was given a 1-point score, and the overall score was obtained by summing up discrete scores for different correct awareness items. Participants with an overall score less than 60% of the total score were considered to have a poor knowledge level, while others with an overall score of 60% or more were considered to have an overall good knowledge level. The descriptive analysis based on frequency and percent distribution was done for all variables: adults' bio-demographic data, education, job title, and osteoporosis history. Also, adults' knowledge and awareness of their daily practice regarding osteoporosis were tabulated with their overall knowledge level, and symptoms of osteoporosis were graphed. Cross-tabulation graphs were used to assess factors associated with adults' overall knowledge level, with significance tested using the Persons' chi-square test and the exact probability test for small frequency distributions.

Ethical considerations

Ethical approval was obtained from the institutional review board of King Abdulaziz University, Jeddah, Saudi Arabia, before data collection (Reference No. 282-23). We included a statement explaining the nature and purpose of the study to obtain participants' consent before they completed the questionnaire. All the data was handled anonymously, saved securely, and used only for research purposes.

## Results

The study comprised a total of 232 eligible adults. Participants’ ages varied from 18 to over 55 years, with a mean age of 35.6 ± 8.7 years. More than half of the participants were female, 132 (56.9%), and out of the total sample, only 14 (6%) were non-Saudi. Regarding marital status, 124 (53.4%) were married, 97 (41.8%) were single, and the rest were either divorced or widowed. The majority of the participants, 147 (63.4%), possessed a bachelor’s degree; 36 (15.5%) even had a postgraduate degree, while the remaining 49 (21.1%) had only a secondary degree. Among the participants, 80 (34.5%) were unemployed, 75 (32.3%) were employed, 11 (4.7%) had private work, and 48 (20.7%) had other work. A monthly income of less than 3000 Saudi Arabian Riyal (SAR) was reported among 88 (37.9%), while 43 (18.5%) had a monthly income of 5000 to 10000 SAR, and 35 (15.1%) exceeded 15000 SAR monthly. Out of the overall sample, 129 individuals (55.6%) reported being diagnosed with osteoporosis or knowing someone who has been afflicted with the condition (Table 1).

**Table 1:** Personal characteristics of study adult populations, Jeddah, Saudi Arabia (n=232)

Personal data	No	%
Age in years		
18-25	86	37.1%
26-35	23	9.9%
36-45	40	17.2%
46-55	43	18.5%
>55	40	17.2%
Gender		
Male	100	43.1%
Female	132	56.9%
Nationality		
Saudi	218	94.0%
Non-Saudi	14	6.0%
Marital status		
Single	97	41.8%
Married	124	53.4%
Divorced/widow	11	4.7%
Educational level		
Secondary/below	49	21.1%
Bachelor’s degree	147	63.4%
Post-graduate degree	36	15.5%
Employment		
Unemployed	80	34.5%
Employed	75	32.3%
Private work	11	4.7%
Retired	18	7.8%
Others	48	20.7%
Monthly income		
< 3000 SAR	88	37.9%
3000-5000 SAR	38	16.4%
5000-10000 SAR	43	18.5%
10000-15000 SAR	28	12.1%
> 15000 SAR	35	15.1%
Have you or someone you know ever been diagnosed with Osteoporosis		
Yes	129	55.6%
No	91	39.2%
I don’t know	12	5.2%

A total of 228 (98.3%) participants heard about the term osteoporosis; 136 (58.6%) think family history can be a risk factor for osteoporosis; and 171 (73.7%) know that osteoporosis is primarily caused by increasing age. Exactly 210 (90.5%) know that vitamin D deficiency is associated with osteoporosis, and 210 (90.5%) say that deficiency of calcium is considered a risk factor for osteoporosis. Exactly 147 (63.4%) think taking calcium in early life can help prevent being diagnosed with osteoporosis. One hundred and eighty-nine (81.5%) think women are at a higher risk of getting diagnosed with osteoporosis than men, 121 (52.2%) think children can get diagnosed with osteoporosis, and 124 (53.4%) think that smoking and alcoholism can lead to the development of osteoporosis. Also, 147 (63.4%) think that other underlying medical conditions can cause osteoporosis. One hundred and forty-one (60.8%) believe that using medications to treat other disorders can lead to osteoporosis, and 179 (77.2%) believe that regular exercise can help prevent osteoporosis. A total of 70 (30.2%), 12 (5.2%), and 50 (21.6%) mentioned estrogen, androgens, or both of them are deficient in patients with osteoporosis, respectively (Table 2).

**Table 2:** Knowledge and awareness of osteoporosis and its risk factors among the adult population in Jeddah, Saudi Arabia

Knowledge and awareness		No	%
Have you ever heard about the term Osteoporosis	Yes	228	98.3%
	No	4	1.7%
Do you think family history can be a risk factor of osteoporosis	Yes	136	58.6%
	No	39	16.8%
	I don’t know	57	24.6%
Osteoporosis is primarily caused due to increasing which of the following	Age	171	73.7%
	Stress	22	9.5%
	Height	4	1.7%
	I don’t know	35	15.1%
Deficiency of which vitamin is associated with osteoporosis	Vitamin D	165	71.1%
	Vitamin A	8	3.4%
	Vitamin B	9	3.9%
	Vitamin C	25	10.8%
	I don’t know	25	10.8%
Deficiency of which mineral is considered as risk factor of osteoporosis	Calcium	210	90.5%
	Magnesium	4	1.7%
	Phosphate	3	1.3%
	I don’t know	15	6.5%
Do you think taking calcium in early life can help prevent being diagnosed with osteoporosis	Yes	147	63.4%
	No	29	12.5%
	I don’t know	56	24.1%
Which hormone is deficient in patients of osteoporosis	Estrogen	70	30.2%
	Androgen	12	5.2%
	Both of them	50	21.6%
	I don’t know	100	43.1%
Do you think women are at a higher risk to get diagnosed with osteoporosis than men	Yes	189	81.5%
	No	13	5.6%
	I don’t know	30	12.9%
Do you think children can get diagnosed with osteoporosis	Yes	121	52.2%
	No	48	20.7%
	I don’t know	63	27.2%
Do you think that smoking and alcoholism can lead to the development of osteoporosis	Yes	124	53.4%
	No	22	9.5%
	I don’t know	86	37.1%
Do you think that other underlying medical conditions can cause osteoporosis	Yes	147	63.4%
	No	10	4.3%
	I don’t know	75	32.3%
Do you think that using some medications for treating some other disorders can lead to the development of osteoporosis	Yes	141	60.8%
	No	12	5.2%
	I don’t know	79	34.1%
Do you think that regular exercises can help prevent osteoporosis	Yes	179	77.2%
	No	17	7.3%
	I don’t know	36	15.5%
Do you think that there is any screening test available for osteoporosis	Yes	180	77.6%
	No	6	2.6%
	I don’t know	46	19.8%

The most reported symptoms recognized by participants were increased fracture frequency (189, 81.5%), stooped posture (100, 43.1%), and height loss (45, 19.4%). Only 38 (16.4%) adults weren't able to identify the common symptoms associated with osteoporosis.

**Figure 1:**
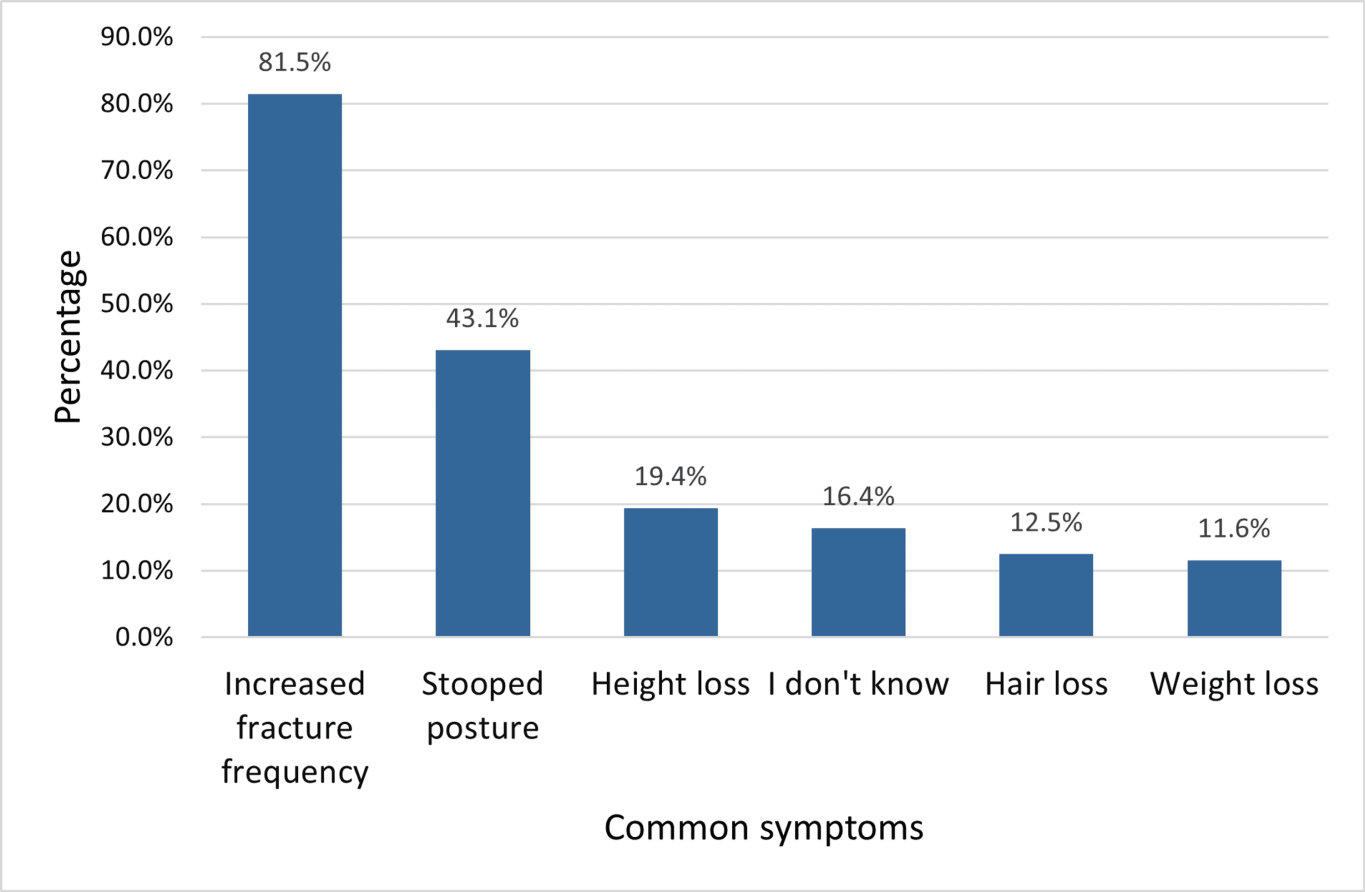
The symptoms of osteoporosis as reported by study adults in Jeddah, Saudi Arabia More than one symptom could be selected

Participants were given the choice to select more than one symptom that can lead to the suspicion of osteoporosis (Figure 1).

**Figure 2:**
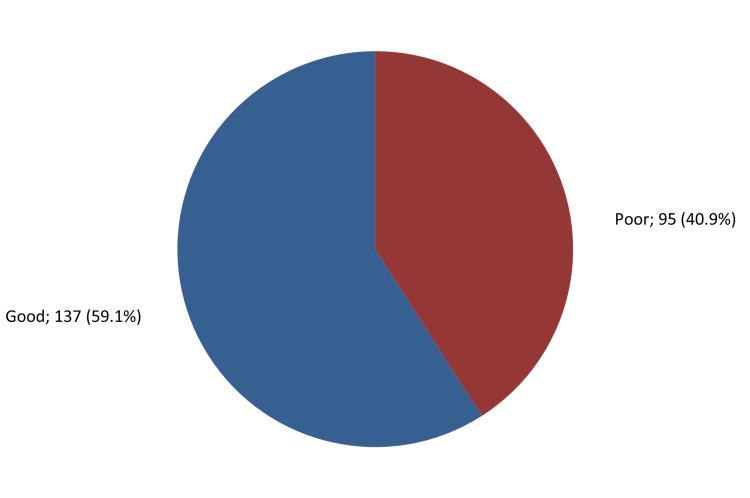
Overall knowledge and awareness level about osteoporosis among adults in Jeddah, Saudi Arabia

A total of 137 adults (59.1%) in the study had overall good knowledge and awareness about osteoporosis, while 95 (40.9%) had a poor knowledge level (Figure 2).

A total of 102 (44%) practiced a healthy and physically active lifestyle only some days; 59 (25.4%) practiced but skipped some days; and 25 (10.8%) always did. Likewise, 82 (35.3%) consumed healthy, nutritious food and dairy products in their routine only some days; 81 (34.9%) did but skipped some days; and 57 (24.6%) always did (Table 3).

**Table 3:** Study adult population practice regarding osteoporosis, Jeddah, Saudi Arabia

Practice items	No	%
Do you practice a healthy and physically active lifestyle?		
Yes, always	25	10.8%
Yes, but skip some days	59	25.4%
Only some days	102	44.0%
I don’t think so	46	19.8%
Do you consume healthy nutritious food and dairy products in routine?		
Yes, always	57	24.6%
Yes, but skip some days	81	34.9%
Only some days	82	35.3%
I don’t think so	12	5.2%

Exactly 63.6% of female adults had an overall good knowledge of osteoporosis versus 53% of male adults, with a recorded statistical significance (p =.049). Also, 77.8% of adults with a post-graduate degree had an overall good knowledge level compared to 55.1% of others with a bachelor's degree and 57.1% of participants with secondary/below education (p =.044). Good knowledge and awareness were detected among 63.6% of adults who were diagnosed with osteoporosis or knew someone who did, compared to 33.3% of those who didn't know and 56% of participants who weren't diagnosed with osteoporosis or didn't know someone who did (p =.045) (Table 4).

**Table 4:** Factors associated with adults' population knowledge and awareness about osteoporosis P: Pearson X2 test, ^: Exact probability test * P < 0.05 (significant)

Factors	Overall knowledge level				p-value
	Poor		Good		
	No	%	No	%	
Age in years					.250
18-25	39	45.3%	47	54.7%	
26-35	12	52.2%	11	47.8%	
36-45	18	45.0%	22	55.0%	
46-55	13	30.2%	30	69.8%	
>55	13	32.5%	27	67.5%	
Gender					.049*
Male	47	47.0%	53	53.0%	
Female	48	36.4%	84	63.6%	
Nationality					.125^
Saudi	92	42.2%	126	57.8%	
Non-Saudi	3	21.4%	11	78.6%	
Marital status					.442
Single	44	45.4%	53	54.6%	
Married	46	37.1%	78	62.9%	
Divorced/widow	5	45.5%	6	54.5%	
Educational level					.044*
Secondary/below	21	42.9%	28	57.1%	
Bachelor’s degree	66	44.9%	81	55.1%	
Post-graduate degree	8	22.2%	28	77.8%	
Employment					.364^
Unemployed	38	47.5%	42	52.5%	
Employed	29	38.7%	46	61.3%	
Private work	6	54.5%	5	45.5%	
Retired	5	27.8%	13	72.2%	
Others	17	35.4%	31	64.6%	
Monthly income					.668
< 3000 SAR	40	45.5%	48	54.5%	
3000-5000 SAR	14	36.8%	24	63.2%	
5000-10000 SAR	18	41.9%	25	58.1%	
10000-15000 SAR	12	42.9%	16	57.1%	
> 15000 SAR	11	31.4%	24	68.6%	
Have you or someone you know ever been diagnosed with Osteoporosis?					.045*^
Yes	47	36.4%	82	63.6%	
No	40	44.0%	51	56.0%	
I don’t know	8	66.7%	4	33.3%	

Of the total adults with good overall knowledge, 12.4% practice a healthy and physically active lifestyle always; 29.2% practice an active lifestyle but skip some days; 43.1% practice for only some days; and 15.3% don't think that they practice a healthy lifestyle (p =.104). In regard to the participants with poor knowledge levels, only 8.4% practice an active lifestyle daily, 20% practice most days, 45.3% only practice some days, and the remaining 26.3% don't think they have a healthy and active lifestyle (p =.104). When comparing the consumption of healthy nutritious food and dairy products in routine in adults with good overall knowledge, 26.3% of them reported always consuming healthy food, 37.2% also routinely consumed healthy diet but skipped some days, 32.1% only consumed them on certain days, and just 4.4% didn't think they consumed a healthy diet (p =.512). The total number of participants with poor overall knowledge who consumed a healthy, nutritious diet, including dairy products, was 22.1%; 31.6% maintained a healthy diet but skipped some days; the majority of 40% had a healthy diet with dairy products only on some days; and the rest, 6.3%, didn't have a healthy diet (p =.512) (Table 5). 

**Table 5:** Relation between adults' knowledge about osteoporosis and their lifestyle practices P: Pearson X2 test, ^: Exact probability test

Practice	Overall knowledge level				p-value
	Poor		Good		
	No	%	No	%	
Do you practice a healthy and physically active lifestyle?					.104
Yes, always	8	8.4%	17	12.4%	
Yes, but skip some days	19	20.0%	40	29.2%	
Only some days	43	45.3%	59	43.1%	
I don’t think so	25	26.3%	21	15.3%	
Do you consume healthy nutritious food and dairy products in routine?					.512^
Yes, always	21	22.1%	36	26.3%	
Yes, but skip some days	30	31.6%	51	37.2%	
Only some days	38	40.0%	44	32.1%	
I don’t think so	6	6.3%	6	4.4%	

## Discussion

Osteoporosis is a global public health concern due to the increased risk of bone fragility and fractures and its associated consequences and burdens on affected populations and the healthcare system [13]. Between 2019 and 2023, the number of fragility fractures in Saudi Arabia has been estimated to be around 988,029, with an associated approximate economic burden of SAR13.5 billion ($3.60 billion United States Dollar (USD); $8.77 billion purchasing power parity (PPP)) in the same timeline [15]. The objective of this study was to evaluate the level of understanding and awareness of osteoporosis and its associated risk factors among the adult population residing in Jeddah, Saudi Arabia. The findings of this study revealed a significant degree of overall consciousness among the participants since the majority (228; 98.3%) were aware of the disorder, and more than half (129; 55.6%) either had personal experience with it or knew someone who had been diagnosed with it. This study's findings aligned with a cross-sectional survey among university students in Jeddah, revealing a high level of overall knowledge on osteoporosis (92%) [16]. A nationwide community-based study done in 2015 reported similar levels of awareness (78%) [17]. Our results showed a better overall knowledge level of osteoporosis, which can contribute to successful public health initiatives focused on osteoporosis, in line with the National Plan for Osteoporosis Prevention and Management. The plan has identified osteoporosis as a national priority and has implemented campaigns like "Immunize your bones" to raise awareness about the condition [18].

One hundred and thirty-seven (59.1%) of participants showed a good overall knowledge level for osteoporosis, with the majority correctly identifying the most important risk factors such as mineral deficiency (210, 90.5%), age (171, 73.7%), and family history (136, 58.6%). A cross-sectional study done in 2016 among 400 female medical school students in Pakistan revealed a relatively lower knowledge of risk factors, contrary to the findings of this study [19]. The study found that a family history of osteoporosis was considered a risk factor by 36.0% of the participants; older age was considered a risk factor by 62.5%; and 47.0% were aware of mineral deficiency, such as calcium, as a risk factor [19]. The results of our study reflected the successful communication of important risk factors for osteoporosis; however, there is a lack of a broader understanding of specific risk factors, such as susceptibility in children, which only 121 (52.2%) identified. These findings are consistent with those of previous studies conducted in this region, which also revealed consistently greater knowledge among the general population. A study by Alamri et al. revealed that 72.6% of respondents had great general knowledge of osteoporosis [17]. Another cross-sectional online survey by Elmorsy et al. found that using the Osteoporosis Knowledge Assessment Tool (OKAT), most participants (84.5%) had moderate-to-good knowledge [20].

However, a study by Khired et al. reported somewhat different findings: among female university students, 16% had knowledge of osteoporosis, which could be attributed to the younger population studied (17-25 years old) [21]. Comparative analysis revealed no significant differences in knowledge scores across demographic variables such as age, sex, nationality, marital status, education, employment status, or income; however, participants who had previously heard about osteoporosis had higher knowledge scores (p = 0.016). In contrast to our study, which did not find an association between age and knowledge of osteoporosis, a previous study by Alqahtani et al. revealed that females aged above 40 had significantly higher knowledge levels of osteoporosis when compared to younger females (aged 18-40) [22]. While our study didn’t show a relationship between age group and knowledge of osteoporosis, 63.6% of participants who were either diagnosed with osteoporosis or knew someone who did have a good overall knowledge level when compared to 33.3% of others who weren't sure they knew someone with osteoporosis and 56% of participants that didn't get diagnosed or knew someone diagnosed with osteoporosis, with statistical significance (p = 0.045). In addition, gender and education level significantly affected the level of knowledge of osteoporosis, as shown in the results of this study. Several other factors, including age, residence, income, occupation, and family history, were identified in studies done in the region as being significantly associated with knowledge about osteoporosis in the general population [22-24], indicating that current public health efforts might involve reaching a diverse population with a basic awareness of osteoporosis and that being aware of the term osteoporosis might increase individuals’ awareness of this topic.

Regarding lifestyle habits, only a small percentage (25, or 10.8%) reported consistently practicing healthy and active lifestyles, and 57, or 24.6%, of the participants consumed healthy foods. Of the 137 (59.1%) adults with a good overall knowledge level, only 17 (12.4%) kept a routine of maintaining a physically active lifestyle, while 36 (26.3%) regularly consumed healthy, nutritious food. This highlights the low level of preventive measures being followed in both groups, adults with good and poor overall knowledge, demonstrating the need for intervention in promoting a healthy lifestyle, and diet as the level of knowledge wasn't a significant factor in assessing the practice of healthy measures. Similar findings have been reported previously. A study by Tripathi et al. reported that although people were aware of the risk factors of osteoporosis, their practices for the prevention of osteoporosis were poor [25]. Similar findings were also reported by Tlt et al., who reported negative attitudes toward screening and the prevention of risk factors [26].

By employing a representative sample size and an online questionnaire, the study effectively reached a diverse population as all age groups and socioeconomic statuses were well represented, reducing the potential biases associated with traditional recruitment methods. This approach facilitated inclusivity and accessibility, ensured diverse participation, and enriched the validity of the study. Our study showed similar results to studies done in the region, proving the significance of socioeconomic factors on the overall knowledge of osteoporosis. 

However, we acknowledge a potential limitation inherent in the reliance on an online questionnaire, as it may inadvertently exclude individuals without Internet access or those lacking technological proficiency. In addition, we weren't able to fulfill the estimated sample size as the response rate was low. Furthermore, we used a convenience sampling method. Finally, self-reported habits may cause underreporting of unhealthy behaviors, affecting the accuracy and completeness of the data collected and the results presented, as it largely rests on the honesty and transparency of the participants. 

## Conclusions

In conclusion, this study provided insights into the knowledge and awareness of osteoporosis among the adult population in Jeddah, Saudi Arabia. The findings indicated a high level of general awareness of osteoporosis among the participants; however, there are still gaps in knowledge, particularly regarding specific risk factors, such as osteoporosis in children. Additionally, there is a need for targeted interventions to promote healthier habits, as only 10.8% of the participants consistently reported practicing a healthy and active lifestyle. Future studies should explore ways to bridge the gap between knowledge and preventive actions, such as promoting healthy lifestyle habits. Furthermore, ongoing campaigns should emphasize the importance of a healthy lifestyle, as well as knowledge about osteoporosis risk factors.

## Appendices

Online questionnaire 

1. Gender

i) Male

ii) Female

2. Age

i) 18-25

ii) 26-35

iii) 36-45

iv) 46-55

v) 55<

3. Nationality

i) Saudi

ii) Non-Saudi

4. Marital status

i) Single

ii) Married

iii) Divorced/Widowed

5. Educational level

i) Secondary/below

ii) Bachelor’s degree

iii) Post-graduate degree

6. What is your current employment status

i) Employed

ii) Unemployed

iii) Private work

iv) Retired

v) Others

7. How much is your monthly income[Saudi Arabian Riyal(SAR)]

i) 3000<

ii) 3000 - 5000

iii) 5000 - 10000

iv) 10000 - 15000

v) 15000<

8. Have you ever heard about the term osteoporosis

i) Yes

ii) No

9. Have you or someone you know ever been diagnosed with osteoporosis

i) Yes

ii) No

iii) I don't know

10. Do you think family history can be a risk factor of osteoporosis

i) Yes

ii) No

iii) I don't know

11. Osteoporosis is primarily caused due to increasing which of the following

i) Height

ii) Age

iii) Stress

iv) I don't know

12. Deficiency of which vitamin is associated with osteoporosis

i) Vitamin A

ii) Vitamin B

iii) Vitamin C

iv) Vitamin D

v) I don't know

13. Deficiency of which mineral is considered as risk factor of osteoporosis

i) Phosphate

ii) Magnesium

iii) Calcium

iv) I don't know

14. Do you think taking calcium in early life can help prevent being diagnosed with osteoporosis

i) Yes

ii) No

iii) I don't know

15. Which hormone is deficient in patients of osteoporosis

i) Estrogen

ii) Androgen

iii) Both

iv) I don't know

16. Do you think women are at a higher risk to get diagnosed with osteoporosis than men

i) Yes

ii) No

iii) I don't know

17. Do you think children can get diagnosed with osteoporosis

i) Yes

ii) No

iii) I don't know

18. Do you think that smoking and alcoholism can lead to the development of osteoporosis

i) Yes

ii) No

iii) I don't know

19. Do you think that other underlying medical conditions can cause osteoporosis

i) Yes

ii) No

iii) I don't know

20. Do you think that using some medications for treating some other disorders can lead to the development of osteoporosis

i) Yes

ii) No

iii) I don't know

21. Do you think that regular exercises can help prevent osteoporosis

i) Yes

ii) No

iii) I don't know

22. Do you practice a healthy and physically active lifestyle

i) Yes, always

ii) Yes, but skip some days

iii) Only some days

iv) I don't think so

23. Do you consume healthy nutritious food and dairy products in routine

i) Yes, always

ii) Yes, but skip some days

iii) Only some days

iv) I don't think so

24. What are the common symptoms that can lead to suspicion of osteoporosis (you can choose more than one)

i) Increased fracture frequency

ii) Weight loss

iii) Height loss

iv) Stooped posture

v) Hair loss

vi) I don’t know

25. Do you think that there is any screening test available for osteoporosis

i) Yes

ii) No

iii) I don't know
